# SYSTEMS-LEVEL MODELING OF BIOLOGICAL AND MOLECULAR AGING CHANGES OVER TIME

**DOI:** 10.1093/geroni/igz038.2145

**Published:** 2019-11-08

**Authors:** Morgan E Levine, Perry Kuo, Jennifer Schrack, Eleanor M Simonsick, Susan Resnick, Michelle Shardell, Luigi Ferrucci

**Affiliations:** 1 Yale University School of Medicine, New Haven, Connecticut, United States; 2 Johns Hopkins, Maryland, Maryland, United States; 3 National Institute on Aging, Baltimore, Maryland, United States

## Abstract

Aging is associated with numerous changes at all levels of biological organization. Harnessing this information to develop measures that accurately and reliably quantify the biological aging process will require longitudinal modeling and incorporation of systems level approaches. We will describe applications of network modeling for longitudinal multi-system biomarker data. Using data from the Baltimore Longitudinal Study of Aging (BLSA) we are able to generate systems level models of biological and physiological function, and then demonstrate how these networks change with age. We will also link systems-level aging changes to hallmarks of aging, including epigenetic alterations, senescence, mitochondrial dysfunction, and proteostasis. Given the complexity of the biological aging process, modeling of systems dynamics over time will both lead to the development of better biomarkers of aging, and also inform our conceptualization of how alterations at the molecular level propagate up levels of organization to eventually influence morbidity and mortality risk.

